# Perceived Stress Scale: Reliability and Validity Study in Greece

**DOI:** 10.3390/ijerph8083287

**Published:** 2011-08-11

**Authors:** Eleni Andreou, Evangelos C. Alexopoulos, Christos Lionis, Liza Varvogli, Charalambos Gnardellis, George P. Chrousos, Christina Darviri

**Affiliations:** 1 Postgraduate Course Stress Management and Health Promotion, School of Medicine, University of Athens, Soranou Ephessiou Str., 4, GR-115-27, Athens, Greece; E-Mails: eandeou@gmail.com (E.A.); varvogli@otenet.gr (L.V.); cdarviri@med.uoa.gr (C.D.); 2 Department of Social Medicine, Faculty of Medicine, University of Crete, Heraklion 74100, Crete, Greece; E-Mail: lionis@galinos.med.uoc.gr; 3 Technological Educational Institute of Messolonghi, 30200 Messolonghi, Greece; E-Mail: bgna@teimes.gr; 4 1st Department of Pediatrics, Athens University Medical School, Athens 11527, Greece; E-Mail: chrousge@med.uoa.gr

**Keywords:** Perceived Stress Scale, translation, psychometric properties, validation, Greece

## Abstract

**Objective:**

To translate the Perceived Stress Scale (versions PSS-4, −10 and −14) and to assess its psychometric properties in a sample of general Greek population.

**Methods:**

941 individuals completed anonymously questionnaires comprising of PSS, the Depression Anxiety and Stress scale (DASS-21 version), and a list of stress-related symptoms. Psychometric properties of PSS were investigated by confirmatory factor analysis (construct validity), Cronbach’s alpha (reliability), and by investigating relations with the DASS-21 scores and the number of symptoms, across individuals’ characteristics. The two-factor structure of PSS-10 and PSS-14 was confirmed in our analysis. We found satisfactory Cronbach’s alpha values (0.82 for the full scale) for PSS-14 and PSS-10 and marginal satisfactory values for PSS-4 (0.69). PSS score exhibited high correlation coefficients with DASS-21 subscales scores, meaning stress (*r* = 0.64), depression (*r* = 0.61), and anxiety (*r* = 0.54). Women reported significantly more stress compared to men and divorced or widows compared to married or singled only. A strong significant (*p* < 0.001) positive correlation between the stress score and the number of self-reported symptoms was also noted.

**Conclusions:**

The Greek versions of the PSS-14 and PSS-10 exhibited satisfactory psychometric properties and their use for research and health care practice is warranted.

## 1. Introduction

Stress refers to the perceived or actual threat on physical and/or psychological homeostasis of the human body [1]. Disrupted homeostasis elicits the so called “stress response”, meaning the activation of central and peripheral neuroendocrine mechanisms responsible for various adaptive responses and behaviors [1]. In the absence of a gold standard measurement of stress, modern scientists adopt three approaches of stress assessment: (1) the environmental approach referring to the occurrence of demanding events (stressors), (2) the psychological approach meaning the perceived by the individual stressfulness of each stressor and (3) the biological approach that focuses on the biological elements of the stress response [2]. Questionnaires and interviews are the main measurement tools of the first two approaches and biomarkers of the biological one. It is thus evident, that public health investigators, who wish to measure stress in large samples and simultaneously maintain accuracy in predicting health related outcomes, need to implement time- and money-saving validated tools of the psychological approach or perceived stress.

There are three popular tools for measuring perceived stress: the Stress Appraisal Measure (SAM), the Impact of Event Scale (IES) and the Perceived Stress Scale (PSS) [3–5]. Among these, PSS is the most widely used such as in studies assessing stressfulness of events, physical and psychiatric diseases and stress management programs [6–14].

PSS was originally developed as a 14-item scale that assess the perception of stressful experiences by asking the respondent to rate the frequency of his/her feelings and thoughts related to events and situations that occurred over the previous month. There are also two product short forms, the PSS-4 and PSS-10 with 4 and 10 respectively selected items by the original PSS-14 form. Notably, high PSS scores have been correlated with higher biomarkers of stress, such as cortisol [15,16]. So far, the scale has been translated in many languages such as Arabic, Swedish, Spanish, Chinese, Japanese and Turkish [17–22]. Very few studies have examined the psychometric properties of the PSS in general population by confirmatory factor analysis (CFA), which is considered as a valid approach to support the construct validity of the PSS [23,24].The aim of this study was to translate and validate this instrument in a Greek community-based sample.

## 2. Methods

### 2.1. Linguistic Validation

After receiving approval from both the Carnegie Mellon University and the authors of PSS (available at www.psy.cmu.edu), we used cross-cultural translation guidelines recommended by International Quality of Life Assessment Project [25] in order to translate the 14 items of PSS from English to Greek. Forward translation was done independently by three bilingual translators and minor differences were solved by the research team. The forward version was then back translated by two other bilingual translators. In a pre-final phase, the first Greek version of the PSS was given to 10 people, who were encouraged to make comments and suggestions on the clarity of the wording, difficulties during completion and on the layout and style of the tool.

### 2.2. Participants and Study Design

The survey took place in hospital wards, public services and universities in four Greek cities (Athens, Thessaloniki, Ioannina, and Rhodes) between October 2009 and April 2010, following a convenient sampling approach. Selected hospitals included Asclepeion Hospital—Voula, Athens; General Hospital of Attica “KAT”; General Hospital of Athens “G. Gennimatas”; Papanikolaou General Hospital—Thessaloniki; Ioannina “Chatzikostas” Hospital and Ioannina University Hospital; and Rhodes General Hospital. Educational institutes included Technological Educational Institute of Athens, the Faculty of Physical Education and Sport Science, University of Athens, ASPETE (Technical & Vocational Teacher Training) Institute; Technological Educational Institute of Thessaloniki and Aristotle University of Thessaloniki. Public services included two Fiscal Services (Financial or Tax Offices) in Athens and one in Thessaloniki and one office of Public Power Corporation in Thessaloniki. In the day of the visit all students sitting at various meeting points (like students clubs, libraries hall) and all employees and visitors at the specific public services and in outpatients clinics of the selected hospitals were asked to participate. Anonymous questionnaires were handed over along with a cover letter explaining the purpose of the study, the researchers’ affiliation and contact information, and clearly stating that the answers would be confidentially treated. Finally, 941 responders (55% response rate) completed the questionnaires comprising of PSS-14, a 21-item translated version of Depression Anxiety and Stress scale (DASS-21) [26] and a symptom check list, which included various stress, anxiety or somatoform-related symptoms [27]. Additional questions on basic demographic data such as age, gender, family, occupational and employment status were also included based on their well-known influence on the perception of stressful experiences.

### 2.3. Instruments Used

#### 2.3.1. Depression Anxiety Stress Scale-21 Items (DASS-21)

The DASS-21 questionnaire is a quantitative measure of distress on the basis of three subscales of depression, anxiety (e.g., symptoms of psychological arousal) and stress (e.g., cognitive, subjective symptoms of anxiety). We used the Greek validated tool of DASS-21 [26]. Each subscale has seven questions that respondents answered according to a Likert-type scale ranging between 0 (“*does not apply to me at all*”) to 3 (“*applies to me very much, or most of the time*”). Although DASS-21 is not a categorical measure of clinical diagnoses, we have used cut-off scores (after multiplying the score obtained by 2 as proposed for comparability with DASS42 full version), which have been developed for defining mild/moderate/severe/extremely severe scores for each DASS scale.

#### 2.3.2. Stress Related Symptoms Checklist

We have used a list of various possibly stress, anxiety or somatoform-related symptoms such as irritability, fatigue, hostility, feeling of tension, inability to concentrate, musculoskeletal symptoms (neck or upper back pain or discomfort), gastrointestinal symptoms (abdominal pain or discomfort, nausea, alterations in bowel habits), headaches, sleep disturbances, tachycardia, increased blood pressure, palpitations, chest discomfort, dizziness and substance abuse [27]. This checklist is not intended as a psychometric tool. It consists of nonspecific symptoms described as related to stress. Stress symptoms, in general, claim more sensitivity than specificity, as such, we were particularly interested on the number of cardinal stress manifestations and not on the evaluation of a situation or psychological state. Participants were asked about the frequency of experiencing these symptoms during the last year and each symptom was binary categorized as frequent or not. Some of these symptoms may not well be expressed as binary variables and suffered low specificity but our interest was to evaluate the coexistence of these stress-related symptoms with high PSS scores. The total number of frequent symptoms was calculated and each participant was categorized in five groups (symptoms less or equal to three, four, five, six and more than six).

#### 2.3.3. PSS Validation

Seven out of the fourteen items of PSS-14 are considered negative (1, 2, 3, 8, 11, 12, 14) and the remaining seven as positive (4, 5, 6, 7, 9, 10, 13), representing perceived helplessness and self-efficacy, respectively. Each item was rated on a five point Likert-type scale (0 = never to 4 = very often). Total scores are calculated after reversing positive items’ scores and then summing up all scores. Total scores for PSS-14 range from 0 to 56 (from 0 to 40 and from 0 to 16, for PSS-10 and PSS-4, respectively). A higher score indicates greater stress.

In order to examine if the Greek version of PSS supports the construct of the two factors of the original PSS, we used confirmatory factor analysis (CFA) on the dataset of the 941 subjects who completed the PSS. Within the structure equation modeling (SEM) procedures in AMOS [28], factorial invariance was examined to see whether the construct is invariant in different groups. A one factor model with all the items as indicators and a two-factor model with items corresponding to the positive and negative factors were fitted to the covariance matrix of the corresponding PSS items. All the CFAs were performed using AMOS SPSS and the maximum likelihood estimation method [28]. Model evaluations were made using a variety of fit indices, including the comparative fit index (CFI) [29], standardized root mean square residual (SRMR) [28], and the root mean square error of approximation (RMSEA) [30]. Values of CFI > 0.9, SRMR < 0.08, and RMSEA < 0.08 are indicative of a good fit with the data [31]. Model chi-square test statistics and associated degrees of freedom and *p*-values were reported for completeness, although they were not used in model evaluation [32]. As a measure of reliability, the internal consistency of the Greek PSS was examined by computing Cronbach’s alpha correlation coefficient for each subscale and for the full scale. Cronbach’s alpha assesses the degree of inter-item correlation and a value larger than 0.70 is considered satisfactory [33]. Concurrent and convergent validity was also evaluated by comparing positive and negative PSS factors with DASS-21 scores and by examining its relation with the number of symptoms, gender and family status. Missing cases did not exceed 2% in any of the comparisons. Cronbach’s alphas, correlation, *t*-tests, Mann-Whitney and Kruskal-Wallis tests as appropriate were computed using SPSS 16.0. All statistical tests were two-tailed, and results were considered significant at *p* < 0.05.

## 3. Results

Among the 941 respondents, most were females (60.5%, *N* = 570), mainly young, with up to 95% of the sample being under 55 years old (total mean age = 29 years old and only 2% above 60 years old), single (55.8%, *N* = 525) and mainly full-time employees (68.7%, *N* = 646), although there were some students (*N* = 232), retired (*N* = 30) and a few unemployed participants.

### 3.1. Confirmatory Factor Analysis

Examination of the fit indexes in Table 1 reveals that the 2-factor models fitted well for PSS-10 and PSS-4 and marginally with PSS-14, while the 1-factor models did not provide acceptable fits except for the PSS-4 version. The standardized factor loadings were presented for all three versions of PSS in Table 2. Overall, standardized factor loadings exceeded 0.4 for PSS-4 and PSS-10. For PSS-14, all loadings exceeded 0.4 except those associated with items 12 and 13 of the negative and positive factor, respectively.

### 3.2. Reliability Analysis

The average inter-item correlations (coefficient alpha values) for the negative subscale were 0.79 for both PSS-14 and PSS-10 (Table 2). For the positive subscales were 0.77 and 0.69 respectively. For PSS-4 version neither positive (0.53) nor negative (0.65) subscale alpha levels exceeded Kline’s criterion of 0.7 for internal consistency [34]. Scores on the positive and negative subscales were computed by averaging the corresponding items for PSS-14 and PSS-10 in each case; higher scores on negative and positive subscales indicate higher levels of perceived distress and coping ability, separately. Overall scores for the three versions of PSS were computed by adding the negative and the reverse of the positive subscale scores. The Cronbach’s alpha values of the full scales were similar (0.82) for PSS-14 and PSS-10, but 0.68 for PSS-4 (Table 2). Both PSS-14 and PSS-10 were tested within the various groups (defined by professional and employment status, town etc.); the differences were very small and in no case found below 0.7 (data not presented for simplicity).

Due to the low internal consistency of PSS-4 in our population and for simplicity, no further results on this PSS version are presented. Table 3 shows statistics on subscale and total PSS scores by gender and family status. Women had significantly higher scores on perceived stress in both subscales and in total, compared to men. Significant differences between divorced or widows with married and singled were found on the scores of PSS questionnaires.

Table 4 shows the means of total and subscale scores on PSS-14 and PSS-10 by the total number of the self-reported symptoms. Those reported no symptoms were few (*n* = 38) and their scores were very close to those reported one to three symptoms, so they reported as one category. A consistent significant trend between the stress score and the number of reported symptoms was identified in sub- and full scales for both PSS versions (all *p*-values smaller than 0.001).

Convergent validity was examined by the correlation of corresponding subscale (stress) of DASS-21 questionnaire. Based on Pearson correlation analysis, PSS-14 was highly correlated with the subscale of DASS-21 for stress (coefficient *r* = 0.644), depression (*r* = 0.606), and anxiety (*r* = 0.542) subscales (all *p*-values smaller than 0.001). Almost identical coefficients were monitored for PSS-10.

According to DASS-21 stress subscale, participants were categorized as having normal or mild level of stress (73%), while 9.4% and 3.3% of them were categorized in the severe and extremely severely affected by stress groups, respectively. In table 5 overall and subscale scores for PSS-14 and PSS-10 are presented across the severity categories of stress subscale of DASS-21 questionnaire. Again the differences between categories were statistically significant (*p* < 0.001).

## 4. Discussion and Conclusions

This study supports the reliability and validity of the Greek version of the Perceived Stress Scale. The validation was based on data provided by 941 urban residents using confirmatory factor analysis. The results show that construct validity, internal consistency and concurrent validity of the Greek version of PSS-14 and PSS-10, and their corresponding subscales were generally supported by our population. As most studies have shown, our findings support a two-factor structure of the PSS-14/-10 versions. On the other hand, results on PSS-4 structure are not consistent. Corroborating these contrary results, we found that PSS-4 version has provided acceptable fits for both one- and two- factor structure [20,23,34]. For PSS-14, two factors loadings of items 12 and 13 were found to be near 0.4 while for PSS-10, Cronbach’s alpha for positive subscale was marginally satisfactory. The relatively low loadings could be due to the translation or the potential interpretation by the subjects which is needed to be verified in further studies utilizing Greek PSS. Further support of the two components in PSS was provided by the opposite correlations of the positive and negative factors while the higher correlation coefficients of “perceived distress” compared to “perceived coping” reflect the assumption that individuals react first to a stressor or threatening event (primary appraisal), and then judge their ability to cope (secondary appraisal) [35]. The internal reliability analysis of the PSS-14 and PSS-10 showed alpha coefficients within the range of other studies and satisfactory. On the other hand the 4-item version yielded a moderate internal consistency and possibly not well acceptable for large scale studies in the general population [33].

As other studies have also shown, females exhibited significant higher stress scores [19,20]. As expected, divorced or widows exhibited higher stress scores compared to singles and especially to married participants.

In addition, we tested the instrument in various strata according to the co-morbidity of stress related symptoms and DASS-21 stress subscale. Our results showed adequate psychometric performance, supporting its use in this population. The increased number of stress related symptoms and the more severe category in DASS stress subscale was strongly and significantly related to increase PSS scores, as expected by the nature of complains and tools [36]. Despite limitations, these findings have implications for cross-cultural research. They support the universality of the conceptual relationships between stress related symptoms, depression, anxiety, and stress as measured with different scales. Furthermore they support the clinical utility of these tools. The Greek versions of PSS-10 and −14 could be used for large scale preliminary screening of stress and to predict the range of health related outcomes in clinical and non-clinical settings presumed to be associated with appraised stress.

The study had several limitations. Generalization about the total population is not warranted due to the opportunistic sampling approach and the heterogeneity of our sample. On the other hand, we believe that this sample would not differ than a random sample from the same population because psychometric properties remained valid and stable in all subgroups analysis. Another limitation is that we didn’t assess test-retest reliability in the current study. Additional population-based studies in various settings on the psychometric properties of the Greek versions of the PSS should be carefully designed and performed to assess concurrent validity and stability of the instrument. In conclusion, the Greek versions of both PSS-14 and −10 showed satisfactory and similar validity and reliability and their use for research and health care practise is warranted.

## Figures and Tables

**Table 1.** Results of confirmatory factor analyses of model testing of PSS-14, PSS-10 and PSS-4.

**MODEL**	**X^2^**	**df**	***p***	**CFI**	**RMSEA**	**SRMR**
PSS-14						
1-factor model	1337.5	78	<0.001	0.633	0.131	0.137
2-factor model	391.0	76	<0.001	0.908	0.068	0.057
PSS-10						
1-factor model	414.7	35	<0.001	0.842	0.107	0.068
2-factor model	165.3	34	<0.001	0.945	0.065	0.041
PSS-4						
1-factor model	39.2	2	<0.001	0.936	0.141	0.045
2-factor model	5.6	1	0.018	0.992	0.070	0.014

CFI = Comparative Fit Index; RMSEA = root mean square error of approximation; SRMR = Standardized root mean square residuals.

**Table 2.** Standardized factor loadings of the 2-factor models fitted to PSS-14, PSS-10 and PSS-4.

**Item**		**PSS-14**		**PSS-10**		**PSS-4**	
		**Negative**	**Positive**	**Negative**	**Positive**	**Negative**	**Positive**
	***In the last month, how often have you (been/felt)***						
1	Upset by something happening unexpectedly?	0.60		0.60			
2	Unable to control the important things in your life?	0.72		0.73		0.73	
3	Nervous and stressed?	0.67		0.66			
8	Could not cope with all the things that you had to do?	0.56		0.57			
11	Angered because of things that were outside your control?	0.54		0.52			
12	Thinking about things that you have to accomplish?	0.35					
14	Difficulties were piling up so high that you could not overcome them?	0.68		0.68		0.67	
4	Dealt successfully with day-to-day problems and annoyances?		0.48				
5	Effectively coping with important changes that were occurring in your life?		0.51				
6	Confident about your ability to handle your personal problems?		0.51		0.48		0.54
7	Things were going your way?		0.61		0.62		0.67
9	Dealt successfully with irritating life hassles?		0.56		0.55		
10	You were on top of things?		0.77		0.78		
13	Able to control the way you spend your time?		0.37				
	Factor correlation	−0.63		−0.66		−0.73	
	Cronbach’s alpha	0.79	0.77	0.79	0.69	0.65	0.53
		0.82		0.82		0.68	

Stress scores by gender, family situation, the number of symptoms and DASS subscales categories (concurrent and convergent validity).

**Table 3.** Total and subscale scores (means and SD) on PSS-14 and PSS-10 by gender and family situation.

	**PSS-14 scores**						**PSS-10 scores**					

	**pos. subscale**		**neg. subscale**		**full scale**		**pos. subscale**		**neg. subscale**		**full scale**	
**Gender**	Mean	SD	Mean	SD	Mean	SD	Mean	SD	Mean	SD	Mean	SD
Men (*n* = 371)	9.94	4.39	13.54	4.89	23.48	7.77	5.92	2.86	10.65	4.47	16.57	6.36
Women (*n* = 570)	10.57	4.46	15.07	4.88	25.64	7.89	6.37	2.89	12.06	4.45	18.44	6.38
p-value *	0.034		<0.001		<0.001		0.019		<0.001		<0.001	
**Age (years)**												
≤ 25 (*n* = 294)	11.34	4.16	14.78	4.76	26.12	7.25	6.73	2.79	11.81	4.31	18.53	6.00
26–35 (*n* = 329)	10.51	4.61	14.52	5.04	25.04	8.18	6.36	2.99	11.54	4.59	17.89	6.63
>35 (*n* = 318)	9.19	4.26	14.13	5.00	23.31	7.99	5.54	2.74	11.19	4.59	16.73	6.51
p-value **	<0.001		0.212		<0.001		<0.001		0.228		<0.001	
**Family situation**												
Married (*n* = 355)	9.34	4.27	13.82	4.87	23.16	7.76	5.61	2.77	10.95	4.51	16.56	6.38
Single (*n* = 525)	10.95	4.46	14.77	4.86	25.72	7.74	6.56	2.92	11.76	4.41	18.32	6.31
Divorced/widow (*n* = 61)	10.69	4.30	15.62	5.60	26.31	8.73	6.41	2.68	12.52	4.96	18.93	6.96
p-value **	<0.001		0.004		<0.001		<0.001		0.011		<0.001	

* Student’s t-test or Mann-Whitney test as appropriate;

** One way ANOVA or Kruskal-Wallis test as appropriate.

**Table 4.** Total and subscale scores (means and SD) of PSS-14 and PSS-10 by the number of stress-related symptoms.

	**Number of symptoms**									
	**≤3 (*n* = 259)**		**4 (*n* = 178)**		**5 (*n* = 232)**		**6 (*n* = 135)**		**≥7 (*n* = 89)**	
**PSS-14 scales**	**Mean**	**SD**	**Mean**	**SD**	**Mean**	**SD**	**Mean**	**SD**	**Mean**	**SD**
Positive	9.27	4.55	9.64	3.95	10.51	4.37	11.22	3.94	12.99	4.68
Negative	12.44	4.60	13.39	4.60	15.07	4.65	15.96	4.62	18.30	4.78
Full	21.71	7.36	23.03	7.04	25.58	7.71	27.19	7.36	31.29	7.85
**PSS-10 scales**										
Positive	5.47	2.93	5.75	2.57	6.35	2.81	6.69	2.57	7.92	3.11
Negative	9.72	4.18	10.49	4.16	11.94	4.26	12.90	4.27	15.07	4.39
Full	15.19	5.97	16.24	5.76	18.29	6.19	19.59	5.99	22.99	6.48

**Table 5.** Overall and subscales scores of PSS-14 and PSS-10 by the level of stress according DASS21 stress subscale.

	**Normal**		**Mild**		**Moderate**		**Severe**		**Extremely Severe**	

	***n* = 567**		***n* = 106**		***n* = 132**		***n* = 88**		***n* = 31**	
**PSS-14 scales**	Mean	SD	Mean	SD	Mean	SD	Mean	SD	Mean	SD
Positive	8.95	4.08	11.17	3.91	12.46	4.04	13.59	3.77	14.68	4.58
Negative	12.50	4.23	15.79	3.74	17.03	4.07	19.38	4.11	21.23	4.52
Full	21.45	6.61	26.96	6.28	29.49	6.45	32.97	5.69	35.90	7.51
**PSS-10 scales**										
Positive	5.29	2.59	6.69	2.42	7.53	2.84	8.32	2.57	9.45	2.93
Negative	9.65	3.83	12.65	3.37	13.92	3.69	16.16	3.57	17.90	3.95
Full	14.94	5.29	19.34	4.96	21.45	5.40	24.48	4.66	27.35	5.88
